# ANCA-related vasculitis incidence and features before and during the COVID-19 pandemic in Los Angeles, Biobio Province, Chile: an observational retrospective analysis

**DOI:** 10.3389/fneph.2025.1599316

**Published:** 2025-06-16

**Authors:** Daniel Enos, Mariel Hernández, Gonzalo P. Méndez, Lysis Cáceres, Ignacia Bravo, Josefina Jobet, Simón Castro, Lorena Cornejo, Catalina Vega, Andrés Salazar

**Affiliations:** ^1^ Nephrology Department, Hospital Victor Rios Ruiz (CAVRR), Los Angeles, Biobio, Chile; ^2^ Internal Medicine Department, Universidad San Sebastián, Los Angeles, Biobio, Chile; ^3^ Internal Medicine Department, Universidad de Concepción, Campus Los Angeles, Los Angeles, Biobio, Chile; ^4^ Internal Medicine Department, Pathology Unit, Laboratorio Inmunocel, Santiago, Chile

**Keywords:** ANCA-associated vasculitis, renal vasculitis, autoimmune diseases, COVID-19, outbreak, renal biopsy, dialysis, mortality

## Abstract

**Introduction:**

Renal vasculitis is a rare disease, the incidence of which increased markedly during the COVID-19 pandemic in our center. The aim of this study is to compare the incidence and the clinical and histopathological characteristics of anti-neutrophil cytoplasm antibodies (ANCA)-associated vasculitis patients before and during the COVID-19 pandemic.

**Methods:**

A single-center observational retrospective analysis of 61 patients with ANCA-associated vasculitis who were divided into two groups according to date of diagnosis: pre-pandemic from 2008 to 2020 (n=37) and during the pandemic from 2020 to the middle of 2022 (n=24). The annual incidence rate was compared, as were characteristics such as age, gender, Birmingham Vasculitis Activity Score (BVAS) score, renal clinic, organ involvement, and ANCA serotype. Biopsy findings, such as optical microscopy glomerular characteristics, crescents, interstitium, immunofluorescence, and electron microscopy findings, were analyzed. Mortality and renal replacement therapy needs were also compared.

**Results:**

The annual incidence rate was higher in the pandemic group compared to the pre-pandemic group, with 9.6 cases per year vs. 3.1 cases per year [incidence rate ratio (IRR)=3.11, 95% CI 1.86 to 5.20]. No significant differences between the groups were found for clinical characteristics, except for greater hemoptysis frequency in the pandemic group. Significant differences in immunofluorescence and electronic microscopy were observed, with a higher IgG deposit and C3 in the pandemic group (37.5% vs 8.1%, p=0.0064; 43.5% vs 10.8%, p=0.009, respectively), whereas the incidence of pauci-immune patterns was higher in the pre-pandemic group (81.1% vs 54.1%, p=0.016). Mortality and the need for renal replacement therapy were significant higher in the pandemic group (IRR=3.56, CI 95% 1.27–9.98 and IRR=4.24, CI 95% 2.08–8.65, respectively)

**Conclusion:**

The incidence of ANCA vasculitis increased during the COVID-19 pandemic and was associated with higher rates of IgG deposit and C3 in the immunofluorescence findings and with higher rates of deaths and dialysis in the pandemic group compared with the pre-pandemic group.

## Introduction

**Figure 1:**
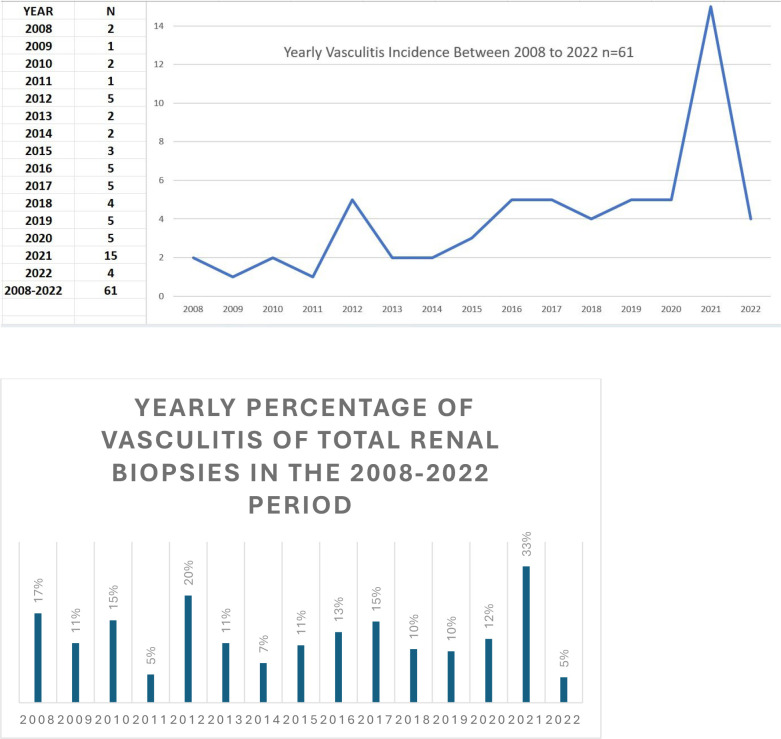
Yearly vasculitis incidence between 2008 and 2022.

Renal vasculitis is an uncommon disease with many improvements in its classification and management over the last two decades (1, 2). As the Kidney Disease Improving Global Outcomes (KDIGO) guidelines state, renal biopsy is desirable in all patients with renal vasculitis (3), helping clinicians to make better clinical-pathologic correlations and select a suitable therapy to avoid further harm. This study was conducted in a south-central Chilean province, with an estimated population of 450,000 inhabitants. As renal vasculitis is a rare endemic disease in our province, in the last 15 years, we have rarely had three to four cases yearly, averaging one case per 100,000 inhabitants in our general hospital. However, this annual incidence significantly increased during the pandemic period (March 2020 to September 2022), in comparison with the 12 previous years, reaching more than eight cases per year, with a remarkable peak in 2021(**Figure 1**). The pathogenesis of viral infection of small vessels remains controversial, with a high probability of a viral triggering of autoimmunity reactions, including post-vaccine vasculitis, because there is evidence of endothelial inflammation and widespread thrombosis in many autopsies of patients infected with SARS-CoV-2 (4–7). The factors behind this increased incidence are not clear, hence our objective was to perform a deeper review of the cases using the biopsy reports first and subsequently examining clinical records, laboratory findings and images, comparing both groups of patients in terms of clinical and histological characteristics to try to identify possible associated factors responsible for the increased frequency and severity.

## Materials and methods

This is an observational retrospective study performed in the Complejo Asistencial Victor Rios Ruiz in Los Angeles, Chile. The trial was approved by the local ethics committee, exempting the requirement of informed consent. The study sample included all patients diagnosed with anti-neutrophil cytoplasm antibodies (ANCA)-associated vasculitis biopsied at our center, based on institutional records. We checked clinical and biopsy reports from 2008 to 2022, including all the cases from that 14-year period. Data were collected throughout 2023, encompassing optical microscopy (OM), electronic microscopy (EN), and immunofluorescence (IF) diagnostic techniques of patients with ANCA-associated renal vasculitis (AARV), all of whom were diagnosed following an indirect immunofluorescence (IFI) positive test and confirmed by ELISA test to be eligible. We divided the patients with AARV into two periods: a pre-pandemic group with 12 years of follow-up (2008–2019) and a pandemic group with 2.5 years (from March 2020 to September 2022), comparing findings and searching for differences between both groups. Herein, we checked the quality of the sample, starting with OM, including the number of glomeruli, percentage of obsolescence, presence or absence of fibrosis, necrosis, crescents (including their three types: cellular, fibro-cellular, and fibrous), vessel damage, interstitial cellularity, and tubular involvement. We subsequently analyzed the immunofluorescence microscopy, looking for the presence or absence of immunoglobulins and complement deposits, before checking the EM findings of each biopsy, looking for deposits, necrosis, endothelium state, basal membrane integrity or rupture, pedicellular state, and Bowman’s space crescents and adherences.

Furthermore, we examined the clinical records of every patient from both groups, starting before the diagnosis and biopsy indication, collecting data on age, gender, and ANCA positivity (all patients were ANCA positive using both IFI and ELISA) and calculating the Birmingham Vasculitis Activity Score (BVAS) at diagnosis to determine the different organs’ involvement and the severity of the illness (8–10). Moreover, we investigated the patients’ need for acute or chronic renal replacement therapy from the first diagnosis and during the follow-up after the biopsy sample until the end of the study. In addition, in the pandemic cohort, we checked for SARS-CoV-2 infection or vaccination prior to AARV diagnosis, investigating the relationship between those facts and vasculitis appearance.

As a result, we compared both populations with renal vasculitis, including clinical and histological findings, using SPSS IBM, making comparisons of interest and using the chi square test for categorical variables with a p-value of 0.05 indicating statistical significance (continuity correction for 2x2 tables, chi square for trend for ordinal variables and Fisher’s exact test in the cases where the assumptions were not met). Means or medians were reported for numerical variables based upon the distribution and were compared using a t-test for those normally distributed and the Mann–Whitney U test for those non-normally distributed. In addition, the rate per 100 person-years was used to assess mortality and renal replacement therapy need, considering the difference between both groups in their follow-up periods. The 95% confidence interval (CI) of the incidence rate ratio (IRR) was calculated.

## Results

The study included 61 patients diagnosed with AARV with a kidney biopsy performed between 2008 and 2022, who were divided into two groups: the pre-pandemic group (n=37) and the pandemic group (n=24). One patient had a non-evaluable biopsy due to inadequate sample quality (only kidney medulla) and was excluded from the histopathological analyses but retained for clinical data analysis.

The median age of each group was 63 and 63.5 years old, respectively, with females being dominant in both groups (59.5% and 62.5%, respectively).

**Table 1** shows the main epidemiological and clinical characteristics of the study population, confirming greater statistically significant vasculitis incidence in the latter, with an annual incidence rate 3.08 cases per year during the first 12 years and 9.6 cases per year between 2020 and 2022 (IRR=3.11, 95% CI: 1.86-5.2), highlighting that after the peak yearly incidence in 2021 the frequency trend mimicked the pre-pandemic period, falling from 15 cases to four in 2022 (**Figure 1**). Even though MPO-ANCA-associated vasculitis was more frequent in both periods, in the pandemic group, the difference was noteworthy but not significant. Minimal differences in the BVAS score (19.2 and 20.2, respectively) and creatinine levels (median 4.65 vs. 4.77) at diagnosis were observed, and neither was statistically significant, with nephritic presentation being predominant in both groups, however, the nephrotic incidence in the pre-pandemic group was not statistically significant (p=0.229). Moreover, we assessed other organ involvement, finding that lungs were most frequently involved without difference between both groups regarding mechanical ventilation need and pulmonary CT scans; however, for hemoptysis, there was a significant difference in favor of the pandemic patients (p=0.045) (**Table 1**).

**Table 1:** Comparison of demographic, clinical, laboratory features, and plasma exchange done between the two vasculitis cohorts: pre-pandemic (2008–2019) and during the pandemic (2020–2022).

Characteristic	Prior to the pandemic (n=37)	Pandemic (n=24)	p-value
Gender, n (%)			1.0
Female	22 (59.5)	15 (62.5)	
Male	15 (40.5)	9 (37.5)	
Age (years)			
Median (IQR)**	63 (47.5–69)	63.5 (55.2–71)	0.451
BVAS score***			
Mean (SD)****	19.2(4.3)	20.2 (5.6)	0.488
Missing (%)	6 (16.2)	2 (8.3)	
Renal clinic (%)			
Nephritic*****	31(83.8)	23 (95.8)	0.229
Nephrotic******	6 (16.2)	1 (4.3)	
Anuric	3 (8.1)	0 (0)	
Hematuria	33 (89.2)	23 (95.8)	0.64
Creatinine median (IQR)	4.65 (2.62–7.77)	4.77 (2.64–8.33)	0.84
Other organ involvement (%)			
LUNG *******	16 (43.2)	14 (58.3)	0.374
Hemoptysis	12 (32.4)	14 (58.3)	0.045*
Mechanical ventilation	5 (13.5)	3 (12.5)	0.909
**Skin**	2 (5.4)	3 (12.5)	0.373
ANCA serology n (%)			
P-MPO	24 (64.8)	21 (87.5)	0.096
C-PR3	13 (35.1)	3 (12.5)	
Plasma Exchange********	10 (27.0)	12 (50.0)	0.068

*p<0.05.

**IQR, Interquartile range.

***Birmingham Vasculitis Activity Score.

****SD, Standard deviation.

***** Less than 2 grams daily.

******Over 3.5 grams daily.

*******CT scan images.

********Available from 2012 in our center.

Furthermore, in terms of mortality, the number of deaths among the pre-pandemic group was 13 across the 12-year period of analysis, whereas in the pandemic group, there were five across the 2.5-year period (2.93 per 100 person-years vs. 8.33 per 100 person-years, IRR=2.85; CI 95% 1.01–7.98, respectively). Renal replacement need was more frequent in the pandemic group, including 11 patients in the 2.5-year-period compared with 24 patients over 12 years in the pre-pandemic group, resulting in a rate of 18.33 dialysis per 100 person-year in the former group as opposed to 5.41 dialysis per 100 person-years in the latter (IRR=3.39; CI 95% 1.66-6.92) (**Table 2**). In relation to the pandemic, only three patients (12.5%) had clinically evident COVID-19 infection prior to the vasculitis onset and, regarding vaccination status, 11 patients (46%) were vaccinated prior to the vasculitis diagnosis, showing a long dispersion time from 42 to 222 days for disease onset after the last shot, with an average of 128 days.

**Table 2:** Death rates and renal replacement need rates in both cohorts.

Outcome	Pre-pandemic (n=37)	Pandemic (n=24)	IRR	CI 95%
Deaths: n (rate per 100 person-years)	13 (2.93)	5 (8.33)	2.85	1.01–7.98
RRT needs: n (RRT per 100 person-years)	24 (5.41)	11 (18.33)	3.39	1.66–6.92

CI, confidence interval; IRR, incidence rate ratio; RRT, renal replacement therapy.

In addition, through the biopsy report analysis, we found no differences between the cohorts in the quality of samples (over 10 glomeruli in 78.5% in the pre-pandemic group and 82.5% in the pandemic group), including other additional glomerular issues such as cellularity (normal or increased), necrosis (present or absent), obsolescence, and fibrosis percentage (<50% or >50%). Additionally, concerning the tubular-interstitial compartment, we looked for sclerosis, cellularity, and edema, without significant differences. After comparing different crescent types in both groups using glomerular surface percentage (cellular, fibro-cellular, and fibrous), there was a non-significant linear trend in the pandemic group towards a higher percentage of fibrous crescents (**Table 3**). Going deeper into the IF results, there were important differences, with greater immunoglobulin G (IgG) and C3 deposits in the pandemic cohort (predominantly in mesangium with an average intensity of 1–2 of 4 crosses, confirmed with electronic microscopy as granular appearance). They were both statistically significant (p=0.0064 and 0.009, respectively). Conversely, the pauci-immune IF was substantially higher in the pre-pandemic group (p=0.016) (**Table 3**). Moreover, 84% in the pre-pandemic group and 87% in the pandemic group had adequate electron microscopy samples, including statistical significant differences in the presence in the pandemic group of distortion of mesangium architecture accompanied by small granular deposits and a thicker basal membrane, consistent with IF findings described above, whereas in the pre pandemic group, the swelling of endothelial cells and basal membrane preservation were significantly different (**Table 4**).

**Table 3:** Compared biopsy findings in patients with renal vasculitis during the pre-pandemic and pandemic periods.

Characteristic	Prior to the pandemic (n=37)	Pandemic (n=23)	p-value
Glomerulus			
Glomerular number per biopsy			0.752
<10	8 (21.6%)	4 (17.4%)	
>10	29 (78.4%)	19 (82.6%)	
Glomerular fibrosis			0.666
<50%	34 (91.9%)	20 (87%)	
>50%	3 (8.1%)	3 (13%)	
Glomerular necrosis			1.0
Yes	25 (67.6%)	15 (65.2%)	
No	12 (22.4%)	8 (34.8%)	
Crescents	37 (100)	23 (100)	
Cellular (%)			0.669
<25	22 (59.5)	14 (56.5)	
25-49	11 (29.7)	5 (21.7)	
50 and more	4 (10.8)	4 (17.4)	
Fibrous-cellular (%)			0.589
<25	27 (73)	15 (65.2)	
25-49	6 (16.2)	5 (21.7)	
50 and more	4 (10.8)	3 (13)	
Fibrous			0.039
<25	29 (78.5)	13 (56.5)	
25-49	6 (16.2)	5 (21.7)	
50 and more	2 (5.4)	5 (21.7)	
Interstitial space			
Sclerosis (%)			0.657
Mild	20 (54.1)	10 (60.9)	
Moderate	13 (35.1)	9 (39.1)	
Severe	4 (10.8)	4 (17.4)	
Increased cellularity (%)			0.417
Mild	6 (16.2)	5 (21.7)	
Moderate	16 (43.2)	11 (47.8)	
Intense	15 (40.5)	7 (30.4)	
Edema			0.840
Mild	11 (29.7)	9 (39.1)	
Moderate	15 (40.5)	6 (26.1)	
Intense	11 (29.7)	8 (34.8)	
Immunoflorescence			
Immunoglobulin A (%)	3 (8.1)	1 (4.3)	1.0
Immunoglobulin G (%)	3 (8.1)	9 (37.5)	0.0064*
C3	4 (10.8)	10 (43.5)	0.009*
Pauci-immune	30 (81.1)	13 (54.1)	0.016*
Linear IgG deposits	1(2.7)	2(8.3)	
*Missing*	0	1	

*p<0.05.

**Table 4:** Comparison of electronic microscopy (EM) findings between both groups.

Characteristic	Pre-pandemic (n=31)	Pandemic (n=21)	p-value
Basal membrane (BM) n (%)			
Preserved BM	25 (81)	10 (48)	0.016^Ϯ^
Thickened BM*	3 (10)	9 (43)	
Thin BM	3 (10)	2 (10)	
Presence of small deposits BM**	1 (1)	2 (10)	
Mesangial (M) n (%)			
Preserved M	17 (55)	8 (38)	0.24°
M distortion***	14 (45)	13 (62)	
M small deposits	4 (13)	9 (43)	0.014°
Endothelial cells (ECs) n (%)			
Preserved ECs	15 ()	13 (62)	0.34°
EC distortion****	16 (52)	8 (38)	
**Presence of crescents, n (%)**	17 (55)	17 (81)	0.01°
Pedicellular involvement n (%)			
Not affected	5 (16)	5 (24)	0.68°
<50%	12 (39)	6 (28)	
Diffuse >50%	14 (45)	10 (48)	
Missing data	*6*	*3*	

*Thickened but no deposits.

**IgG linear deposits.

***Detritus, granular small deposits, and necrosis.

****Swollen and necrotic cells.

°Chi-square test.

ϮFisher´s exact test was applied since assumptions for the chi-square test were not met.

## Discussion

This observational study depicts an outbreak of AARV during the pandemic period, with an annual incidence rate 3.1 times higher than the previous 12 years. Even though there are some reports regarding the relationship between the pandemic period and AARV, they are only isolated or no more than a few cases per report. These include cases reporting COVID-19 clinical symptoms or almost immediately after the clinical picture such as an Euvas analysis of vasculitis triggered after COVID-19 disease or vaccination (11), with some reports of vasculitis reported after clinical COVID-19 infection across wide age ranges, in both male (12, 13) and female patients (4, 5, 14) or following the vaccination shots after the first dose (6, 7, 14) or second dose (15) of the Pfizer-BioNTech vaccine. Furthermore, there are two reports of vasculitis after the ChAd vaccine (16, 17) and finally two reports of three patients with cutaneous vasculitis (18, 19), including 38 patients collected from other reports and analyzed in a literature review (20).

In our analysis, despite many clinical features between both groups being found to be similar, the pandemic group was associated with significantly worse outcomes, such as mortality and renal replacement need, with deaths increasing 2.9-fold and the dialysis requirement increasing 3.4-fold, indicating the severity of the AARV outbreak during the pandemic period.

We think that our findings from the IF of the biopsies are of interest given that it is classically thought that ANCA vasculitis will show in a high proportion of cases, lacking immune deposits as had occurred in most of the pre-pandemic biopsies, with more than 80% with the pauci-immune pattern confirmed with the absence of deposits in EM. Nonetheless, as some authors described years ago, IgG, complement, and other immunoglobulin deposits can be found in AARV (20, 21), reaching up to 45% of IgG deposits in our pandemic group, including the very uncommon linear IgG pattern (22). This may suggest a different pathogenesis of this complex disease during that lockdown time. Moreover, after analyzing the significant C3 deposits in the pandemic group, we think that it could be useful to consider prescribing a C5 complement blocker as a side therapy (23–25), especially when the complement has an important presence in glomerular IF, however, this was not possible for us because it is not available in Chile.

In an effort to address the reasons for this dramatic rise and severity of AARV incidence during the pandemic period, we found that the relationship between a SARS-CoV-2 clinical infection or vaccination prior to the vasculitis onset was not consistent, as only a few of the 24 patients had been ill with a proven SARS-CoV-2 disease and less than 50% received prior vaccination, with vasculitis appearance on average 128 days after the dose, which seems too long a period to be relevant.

Several other factors could explain our findings: one of them could be a link with this pandemic outbreak, and the strict isolation and lockdown regime imposed by our public health authorities in Chile for more than 2 years from March 2020 to October 2022, because it is well-known that innate immune responses are regulated primarily by microorganisms and cell death stimuli, yet they can also be stressed by endocrine and neural systems (25, 26). Beyond this point, we think that it could be probable that a prolonged isolation period may modify these relationships since the immune system produces important neuro-endocrine feedback through many cytokines and could influence physiological, social, and environmental aspects in human wellbeing, indicating a real extrinsic regulatory relationship between both systems (25, 26). This neuro-immune circuit works in both directions, incorporating adaptive and innate immune responses, with both mechanisms possibly playing a role in the central nervous system and immune system to produce autoimmune diseases. Nonetheless, these complex mechanisms are mediated by receptors from the autonomic sympathetic system by releasing different classes of neuroeffectors, allowing them to adapt to environmental changes (26). Thus, it is possible that the great stress caused by the long quarantine period during the pandemic could have produced a change in both innate and adaptative immune responses, leading to a higher incidence of autoimmune diseases and interfering with the hypothalamus-hypophysis-adrenal axis, which can regulate the increase or decrease in glucocorticoids plasmatic levels, the effect of which is an upregulation or suppression of many immunomodulator genes and their derived cytokines, which could stop or develop new diseases respectively, depending on the mental status of the patient. Moreover, many authors correlate psychological stress, including isolation and loneliness, with a rise in pro-inflammatory molecules, despite high glucocorticoid levels, because of the existence of a blunted effect on monocytes, leading to steroid hormone resistance while enhancing Kappa B signaling (26–30), thus producing a greater immune response against their own antigens. Delving deeper into our pandemic patients, the loneliness experienced due to lockdown could have provoked extreme weakness in their immunity, leading to a higher predisposition to more severe viral clinical scenarios, enhancing the inflammatory response as was previously described, and provoking autoimmune diseases (29). However, there were some peculiar aspects to our pandemic lockdown related to a non-voluntary continuous stress of isolation in an environment of fear of death, in addition to bad lifestyle habits, principally with excessive fast-food diets and reduced physical activity to avoid getting infected, added to the overlapping natural feelings of missing relatives and friends (29). In addition, other authors have stated that some proven relationships between adverse life circumstances, such as pandemic lockdowns, and isolation can produce an upregulation of pro-inflammatory genes accompanied by downregulation of interferon and IgG production, leading to immunity disturbances (29). These changes are possible because of an increased transcription of these genes from the leucocytes of isolated people, including monocytes, B and T lymphocytes, and natural killer cells (29, 30).

Finally, all these psychological and biological events could have occurred in our patients with vasculitis in the pandemic group and require further investigation to address the real contribution of each factor to the increased frequency of developing this uncommon disease during this period. It must be remarked that, in 2022, the vasculitis incidence had a downward trend similar to the pre-pandemic period (**Figure 1**), coinciding with the end of quarantine in Chile. A necessary further follow-up may demonstrate the real significance of this period ending and may provide us with some answers that may not currently seem clear at all.

### Teaching points

1. During the pandemic, we witnessed an outbreak of AARV with greater severity, dialysis need, and mortality.
2. The renal biopsy findings in the pandemic cases showed a greater presence of mesangial deposits of IgG and C3, with fewer typical pauci-immune patterns.

### Limitations

1. We have selection bias because, during the pandemic, we lost two severely ill patients who had a short time in the ICU before dying, whose diagnosis was made after they passed away without performing a biopsy, and four patients from the pre-pandemic group were in the same situation. Furthermore, another female patient with ANCA-associated vasculitis did not allow us to take a biopsy in the pandemic group.
2. Given the fact that our study was an observational study, our findings only show the association between clinical variables and do not imply causality in any form, hence, it was not possible to confirm a reason for the increased number of cases of vasculitis during the pandemic, especially during 2021. However, the theories we explored are possible explanations that need further investigation.

## Data Availability

The raw data supporting the conclusions of this article will be made available by the authors, without undue reservation.
